# Setting Physical Activity Goals with a Virtual Coach: Vicarious Experiences, Personalization and Acceptance

**DOI:** 10.1007/s10916-022-01899-9

**Published:** 2023-01-30

**Authors:** Nele Albers, Beyza Hizli, Bouke L. Scheltinga, Eline Meijer, Willem-Paul Brinkman

**Affiliations:** 1 Intelligent Systems, Delft University of Technology, Delft, The Netherlands; 2 Biomedical Signals and Systems, University of Twente, Enschede, The Netherlands; 3 Public Health and Primary Care, Leiden University Medical Center, Leiden, The Netherlands

**Keywords:** Goal-setting, Physical activity, Behavior change, Conversational agent, Self-efficacy, Motivation

## Abstract

Goal-setting is often used in eHealth applications for behavior change as it motivates and helps to stay focused on a desired outcome. However, for goals to be effective, they need to meet criteria such as being specific, measurable, attainable, relevant and time-bound (SMART). Moreover, people need to be confident to reach their goal. We thus created a goal-setting dialog in which the virtual coach Jody guided people in setting SMART goals. Thereby, Jody provided personalized vicarious experiences by showing examples from other people who reached a goal to increase people’s confidence. These experiences were personalized, as it is helpful to observe a relatable other succeed. Data from an online study with a between-subjects with pre-post measurement design (n=39 participants) provide credible support that personalized experiences are seen as more motivating than generic ones. Motivational factors for participants included information about the goal, path to the goal, and the person who accomplished a goal, as well as the mere fact that a goal was reached. Participants also had a positive attitude toward Jody. We see these results as an indication that people are positive toward using a goal-setting dialog with a virtual coach in eHealth applications for behavior change. Moreover, contrary to hypothesized, our observed data give credible support that participants’ self-efficacy was lower after the dialog than before. These results warrant further research on how such dialogs affect self-efficacy, especially whether these lower post-measurements of self-efficacy are associated with people’s more realistic assessment of their abilities.

**Supplementary Information:**

The online version contains supplementary material available at 10.1007/s10916-022-01899-9.

## Introduction

Lack of physical activity is one of the primary risk factors for cardiovascular disease, which causes an estimated 32% of deaths worldwide [1]. To support people in becoming more physically active, goal-setting is commonly used as it provides motivation and helps to stay focused on a desired outcome [2, 3]. As such, goal-setting is often part of eHealth applications (e.g., [4]), which have the potential to make behavior change support more effective and widely available [5–7]. However, for goals to be effective, they need to meet several criteria such as being aligned with other goals a person has [8] and being realistic and achievable [9].

We thus wanted to incorporate the setting of effective goals in eHealth applications for behavior change. To this end, we designed a goal-setting dialog for running or walking with the virtual coach Jody, as virtual coaches can provide guidance where traditionally therapists would have. Virtual coaches can also foster adherence to eHealth applications by improving engagement and connecting with users [10]. In the goal-setting dialog, Jody asked users several questions to encourage the creation of SMART goals [11], which are goals that are specific, measurable, attainable, relevant, and time-bound. For example, Jody asked users why their goal was important to them based on a set of questions therapists use to find out what matters to their clients in their life [12]. Being helped to realize the reasons for wanting to achieve an outcome has previously been shown to lead to more personalized goals and better results [13].

Effective goals alone are, however, not sufficient for behavior change to be successful. This is because there is a variety of possible barriers relating to users’ capability, opportunity, and motivation [14], such as a lack of self-efficacy [2, 15, 16]. One way to increase users’ self-efficacy is to let them observe another person succeed [15], or, in other words, make vicarious experiences. According to social cognitive theory [17], there are four sources of self-efficacy for a behavior: mastery experiences, vicarious experiences, verbal persuasion, and somatic and affective states. Comparing these sources of self-efficacy, Warner et al [18] found that mastery experiences, vicarious experiences, and subjective perceptions of health had similar significant effects on exercise self-efficacy in a study with older adults. The meta-analysis by Ashford et al [19] further identified feedback on past or others’ performance as producing the highest levels of self-efficacy in the context of promoting lifestyle and recreational physical activity, followed by vicarious experiences. Since goal-setting typically first takes place at the start of a behavior change intervention when people may not yet have any personal experience with the target behavior, using vicarious experiences to foster self-efficacy is intuitively appealing.

While it can be helpful to observe another person succeed, it matters how relatable this “other” is: a more relatable “other” is beneficial [15, 20]. Finding such a relatable “other” can be difficult in practice for underserved groups [21] such as older adults [18]. Given vicarious experiences from diverse people, behavior change applications can, however, make use of user data to find relatable others. In our goal-setting dialog, Jody, therefore, showed *personalized* examples of other people who had achieved a running or walking goal.

To evaluate this dialog, we conducted a study in which 39 people set a running or walking goal with Jody. Based on this study, we tested the following hypotheses:

- H1: People’s self-efficacy is higher after the dialog with the virtual coach than before.
- H2: People’s self-efficacy is higher after receiving personalized examples than after receiving generic examples.
- H3: The personalized examples are perceived as more motivating than generic examples.
- H4: People have a positive attitude toward the virtual coach.

In addition, we inquired what people found motivating about the examples from other people.

## Materials & methods

We conducted our study in March 2022. The Human Research Ethics Committee of Delft University of Technology approved the study (Letter of Approval number: 1707), and we preregistered the study in the Open Science Framework (OSF) [22].

### Study design

The study was set up as a mixed-design study with one between- and one within-subject factor. The between-subject factor was the type of examples shown (2 levels: personalized/generic), and the within-subject factor was time (2 levels: pre-/post-measurement).

### Materials

We used the Qualtrics platform to host the online questionnaires and a Google Compute Engine to host the virtual coach.

#### Virtual coach

The virtual coach Jody was implemented in Rasa [23]. It introduced itself as being there to help users set a goal for becoming more physically active. After describing the benefits of physical activity and goal-setting, Jody asked users to provide an initial idea for their choice of either a running or a walking goal. This was to reduce the anchoring effect [24] of the examples from other people. Next, users were shown two examples from other people and subsequently asked to re-formulate their goal as specifically as possible. Afterward, users were asked to reflect on the relevance and attainability of their goal as well as to provide a deadline for reaching their goal. In case of very low or very high attainability, users were suggested to adapt their goal to make it less or more challenging. Finally, Jody summarized users’ goals, upon which they could confirm their goal or change the behavior or its deadline. To increase the accessibility of the dialog, users could largely communicate by clicking on buttons with answer choices. A psychologist read through the dialog to ensure that the language and dialog structure were easy to follow. The implementation of the virtual coach [25] as well as a video of the dialog [26] can be found online.

#### Examples from other people

**Fig. 1:**
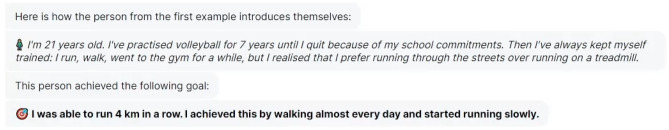
Screenshot of part of the dialog showing an example of a person who achieved a running goal

Each participant saw 2 out of 72 examples from other people. In a prior study, these examples were collected from 72 people, 4 each for 18 combinations of values for sex, age range, and weekly exercise amount. These people from the prior study were asked to 1) introduce themselves to a new person they meet at a friend’s gathering who is interested in their physical activity behavior, 2) describe a goal with regards to running or walking they achieved in the past year, and 3) describe how they achieved that goal. The latter was included because it can be motivating to read not only that somebody else achieved a goal but also how they managed to do so [27]. After the prior study, the collected examples were anonymized and corrected regarding spelling, punctuation, and grammar to allow them to be displayed in the goal-setting dialog. Fig. 1 shows how an example was presented in the dialog. The characteristics of the people who provided the examples are presented in Table 4 and all examples are available online [28].

##### Personalized Examples

**Fig. 2:**
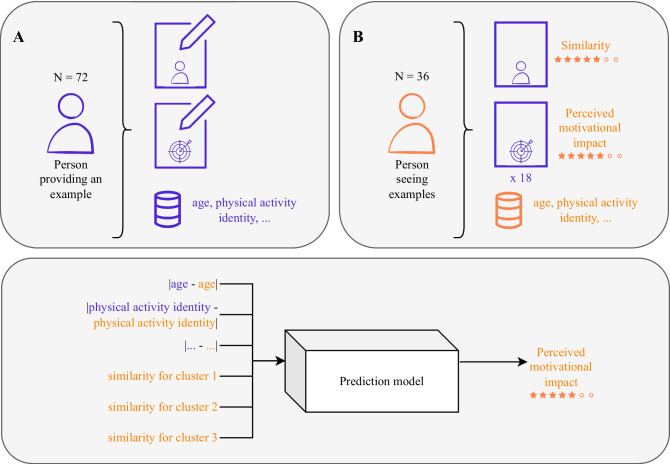
Process of obtaining the prediction model used for choosing personalized examples. **A** 72 example people wrote about themselves and a goal they achieved. **B** 36 people each rated 18 examples on similarity and perceived motivational impact. The results from **A** and **B** were used to obtain the prediction model

People in the “personalized” condition saw examples that were predicted to be most motivating by a linear regression model. To create this model, we conducted a second data collection study on Prolific in which 36 individuals each rated 18^1^ examples on how similar to themselves they considered the corresponding person and how motivating they considered the goal and how the person achieved it. Afterward, the prediction model was set up to predict the motivation rating based on the absolute difference in individual characteristics between the person providing an example and the person seeing an example (Fig. 2). We chose 10 out of 22 variables associated with the predictors of behavior capability, opportunity, and motivation [29] using stepwise regression and correlation analysis (Table 1)^2^. Furthermore, as not only *actual* but also *perceived* similarity can play a role [30], we used as three additional prediction variables the similarity ratings for three clusters of people from the examples based on two prototype examples per cluster. The three clusters were computed based on the similarity ratings and the prototypes were the two most centered examples per cluster. These prototypes were not shown in the goal-setting dialog. The full model had a multiple $R^2$ of 0.23 and is shown in Table 1.

**Table 1:** Multiple linear regression model used to predict motivation ratings

Variable	Estimate	Beta	Std. Error	t value	Pr($>\vert t\vert$)
Individual characteristics					
Age	-0.65	-0.08	0.28	-2.33	0.02*
Extraversion	-0.73	-0.08	0.33	-2.25	0.03*
Openness to experience	-0.79	-0.09	0.31	-2.58	0.01*
Household income	0.77	0.08	0.36	2.11	0.04*
Household size	0.61	0.07	0.33	1.84	0.07
Physical activity identity	-0.44	-0.05	0.39	-1.13	0.26
Sitting hours weekend day	0.57	0.06	0.37	1.55	0.12
TTM-stage	-0.53	-0.08	0.27	-2.00	0.05*
Godin leisure time activity level	-0.89	-0.20	0.18	-5.03	<0.001***
Running/walking self-efficacy	-0.82	-0.10	0.30	-2.72	<0.01**
Similarity ratings					
Similarity rating for cluster 1	0.13	0.09	0.05	2.43	0.02*
Similarity rating for cluster 2	-0.01	-0.01	0.05	-0.20	0.84
Similarity rating for cluster 3	0.40	0.25	0.06	6.79	<0.001***

*TTM* Transtheoretical Model

Signif. codes: 0 ***0.001 **0.01 *0.05

##### Generic Examples

Using the same motivation ratings as in the “personalized” condition, people in the “generic” condition received a random selection of two of the three overall most motivating examples (Table  6).

### Measures

#### Running or walking self-efficacy

These were measured based on scales from 0 to 100, adapted from the Exercise Self-Efficacy Scale by McAuley [31] (see Online Resource 1). 

#### Perceived motivational impact of examples

Participants rated examples from other people on how motivating they perceived them on a scale from -3 to 3. 0 was labeled as “Neutral”. 

#### Acceptance of the virtual coach

We used an adaptation of the six questions by Provoost et al [32], each of which was rated on a scale from -3 to 3, with 0 labeled as “Neutral.” 

#### Take-away from examples

Participants provided a free-text response to the question “What can you take away from these examples for yourself?” after seeing the two examples from other people in the dialog. 

#### Motivational factors from examples

Participants gave a free-text response to the question “What do you find motivating about the running or walking goals that other people achieved?” 

#### Transtheoretical Model (TTM)-stage for becoming physically active

Using the World Health Organization’s definition of physical activity [33] and guidelines on physical activity and sedentary behavior [34], we adapted the question by Norman et al [35] to physical activity to measure this stage of change.

### Participants

**Fig. 3:**
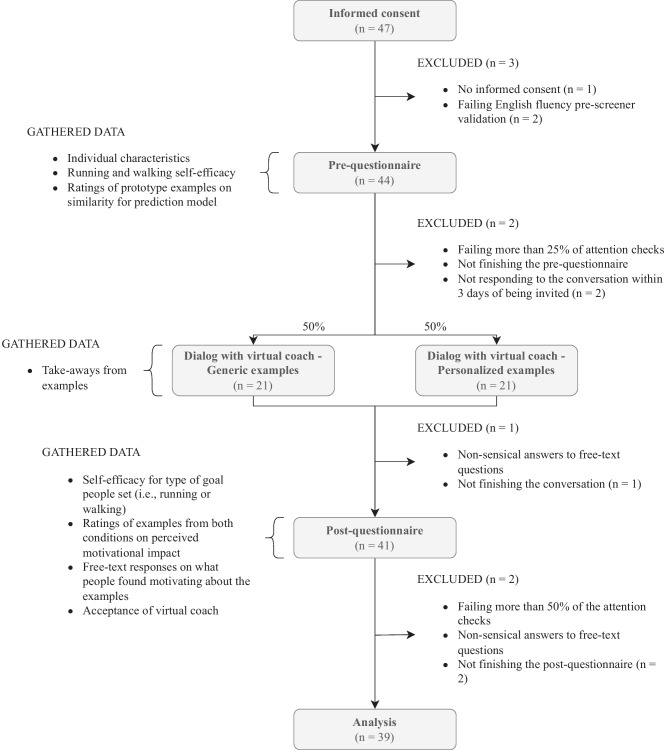
Study design including study components, gathered data, and exclusion criteria

We aimed for a sample size of 36 based on 1) a power analysis using G$^*$Power 3.1 [36] for ANOVA with repeated measures and within-between interaction leading to a sample size of 34 for a power of 0.8, an effect size of 0.25 (i.e., a medium effect size for ANOVA [37]), and an alpha of 0.05 and 2) wanting to recruit participants from 36 combinations of values for the variables smoking status^3^, sex, age, and weekly exercise amount. Eligible were people who were fluently English-speaking adults and who had not participated in the earlier data-gathering studies. 47 participants started the study and 39 were included in the analysis. Exclusion criteria are shown in Fig. 3. Participants were paid based on the minimum payment rules on Prolific (i.e., five GBP/hour).

Participant characteristics are shown in Table 2. Comparing Bayesian models with and without the condition as a predictor for each characteristic did not indicate systematic differences between the conditions for these characteristics.

**Table 2:** Participant characteristics for the two conditions

Characteristics	Generic	Personalized
Number		
- n	20	19
Age		
- mean (SD)	44.4 (17.7)	38.7 (14.6)
Sex		
- Female, n (%)	10 (50.0%)	10 (52.6%)
- Male, n (%)	10 (50.0%)	9 (47.4%)
Godin leisure time activity		
- mean (SD)	36.4 (31.7)	31.0 (22.3)
Running/walking self-efficacy$^*$		
- mean (SD)	74.1 (30.5)	65.5 (24.0)
Sitting hours weekend day		
- mean (SD)	8.6 (3.7)	6.8 (4.3)
Smoking frequency		
- Less than once per day, n (%)	11 (55.0%)	13 (68.4%)
- At least once per day, n (%)	9 (45.0%)	6 (31.6%)
TTM-stage for becoming physically active		
- Precontemplation, n (%)	3 (15.0%)	0 ( 0.0%)
- Contemplation, n (%)	3 (15.0%)	8 (42.1%)
- Preparation, n (%)	6 (30.0%)	3 (15.8%)
- Action, n (%)	2 (10.0%)	3 (15.8%)
- Maintenance, n (%)	6 (30.0%)	5 (26.3%)
Weekly exercise amount		
- Less than 60 minutes per week, n (%)	8 (40.0%)	6 (31.6%)
- 60–150 minutes per week, n (%)	6 (30.0%)	6 (31.6%)
- More than 60 minutes per week, n (%)	6 (30.0%)	7 (36.8%)

*SD* Standard deviation, *TTM* Transtheoretical Model

*Running and walking self-efficacy were measured on scales from 0 to 100

### Procedure

The study consisted of 1) a pre-questionnaire, 2) a goal-setting dialog with the virtual coach in which people saw either generic or personalized examples, and 3) a post-questionnaire (Fig. 3). Participants who successfully completed the pre-questionnaire were invited to the dialog about one week later. These participants were randomly assigned to the two conditions for the examples shown in the goal-setting dialog. Using an adaptation of the algorithm by Xiao et al [39], we aimed to balance the two conditions with regard to smoking status, self-efficacy for the preferred goal type, and two ratings of perceived similarity for clusters of people from the examples. The dialog lasted about seven minutes, after which participants were asked to complete the post-questionnaire.

### Data preparation and analysis strategies

We created two index measures for the ratings of example types (i.e., personalized or generic) on perceived motivational impact as well as an index measure for the acceptance (Cronbach’s $\alpha$ of 0.57, 0.56, and 0.87). Moreover, we reversed the scale of the TTM-stage for becoming physically active such that a higher value denotes a higher stage of change.

Instead of a frequentist analysis, we conducted a Bayesian analysis as it has been described as providing richer information [40]. We used Bayesian paired *t*-tests on the pre- and post-measurement of self-efficacy for H1 and the perceived motivational impact ratings for the two types of examples for H3. For H2, we used a Bayesian two-sample *t*-test on the change in self-efficacy between the pre- and post-measurement, and for H4 a Bayesian one-sample *t*-test comparing the acceptance to a neutral value of zero. We evaluated the posterior probabilities that the hypotheses are true using the guidelines by Chechile [41] and their extension to probabilities below 0.5 by Andraszewicz et al [42]. These guidelines frame posterior probabilities as “bets” one can place with varying levels of confidence. The analyses were conducted in R with the Bayesian First Aid package [43].

For exploratory purposes, we additionally used the Bayesian First Aid package to compute the Pearson correlations between the TTM-stage for becoming physically active on the one hand and the change in self-efficacy between the pre- and post-measurement, motivational impact ratings for the two types of examples, and the acceptance on the other hand.

We further conducted a qualitative analysis of people’s take-aways from the examples they saw as expressed in the dialog and what they found motivating about the examples as described in the post-questionnaire. Triangulating multiple sources serves to increase the validity of qualitative research [44]. Following the thematic analysis steps by Braun and Clarke [45], BH first familiarized herself with the data before creating a draft coding scheme. She discussed the coding scheme with NA to arrive at a final coding scheme with nine codes. BH then coded all responses, using multiple codes if relevant. We found substantial agreement based on double-coding (Cohen’s $\kappa$ = 0.79) [46]. The responses from one participant were afterward excluded from further analysis because the participant shared sensitive information. The codes assigned by BH were subsequently used to identify themes.

All data and analysis code are available in the online repository accompanying this article [47].

## Results

**Fig. 4:**
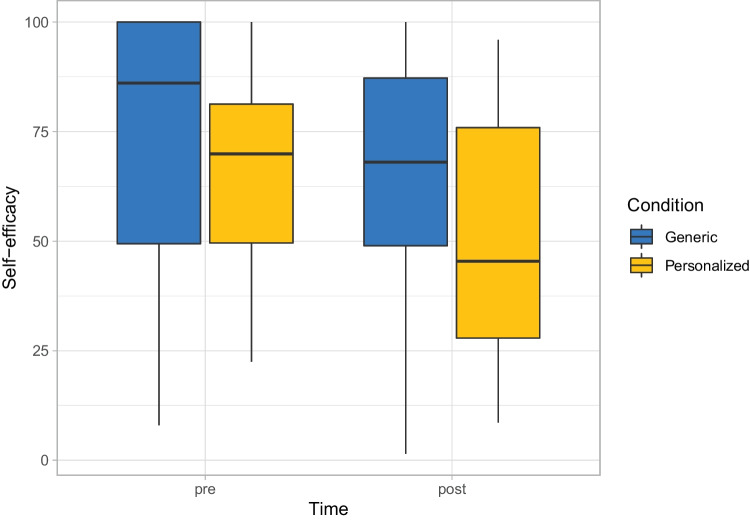
Self-efficacy before and after the dialog with the virtual coach for both conditions

Figure 4 shows that participants on average had a lower self-efficacy after than before the dialog. Quantifying this through our Bayesian analysis shows that the mean drop in self-efficacy was 12.38, which leads to a posterior probability of 0.002 that the self-efficacy is higher after the dialog than before (Table 3). This can be evaluated as a very strong bet against H1. Moreover, contrary to H2, Fig. 4 does not show a higher but rather a somewhat lower self-efficacy in the “personalized” condition compared to the “generic” condition. Our Bayesian analysis suggests that it is not worth betting against H2 based on a posterior probability of 0.34 that H2 is true (Table 3). The perceived motivational impact of the personalized examples is, however, by on average 0.31 scale points higher than the one of the generic examples (Table 3). This leads to a posterior probability of 0.93 that H3 is true, which can be evaluated as a promising but risky bet. Regarding H4, the mean for people’s acceptance of Jody is 1.54, with the corresponding 95% credible interval ranging from 1.19 to 1.90 (Table 3). Based on a posterior probability of >0.99995, it is virtually certain that H4 is true.

Our exploratory analysis further provides no strong indication for correlations between the TTM-stage on the one hand and the change in self-efficacy and the motivational impact ratings for the two types of examples on the other hand (Table 7). For the acceptance, however, we see a small correlation [48] of 0.22 with a corresponding posterior probability of 0.92 that the correlation is greater than 0. Thus, we obtain a promising but risky bet that people in higher stages of change have a more positive attitude toward Jody.

**Table 3:** Results of Bayesian analyses for the four hypotheses

Parameter	Mean (SD)	95% CI	Post	Evaluation
H1: Self-efficacy				
Post - Pre	-12.38 (24.54)	[-20.36, -4.15]	0.002	Very strong bet against
H2: Change in self-efficacy				
Personalized - Generic	-3.57 (1.36)	[-21.03, 13.46]	0.34	Not worth betting against
H3: Perceived motivational impact				
Personalized - Generic	0.31 (1.19)	[-0.09, 0.72]	0.93	A promising but risky bet
H4: Acceptance				
Mean	1.54 (1.07)	[1.19, 1.90]	>0.99995	Virtually certain

*SD* Standard Deviation, *CI* Credible Interval, *Post* Posterior probability that the hypothesis is true

**Fig. 5:**
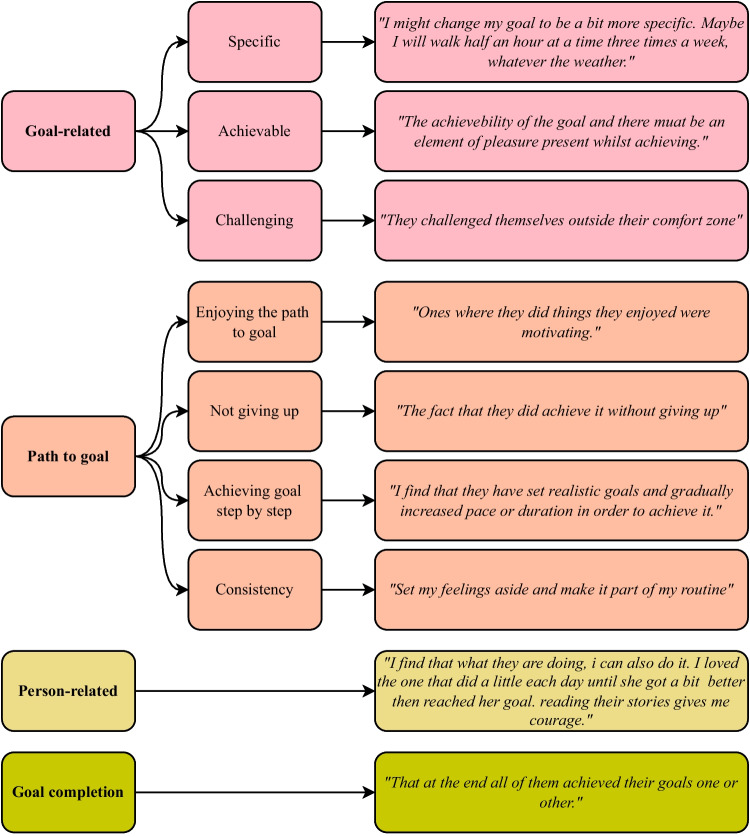
The four themes for what people found motivating about examples from other people with sub-themes and participant quotes

**Fig. 6:**
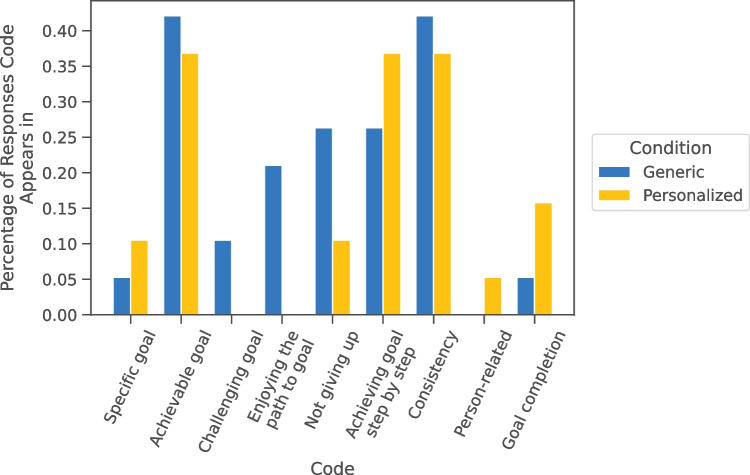
Percentage of take-away responses each code from the coding scheme appears in per condition

Figure 5 shows the four themes for what people found motivating about examples from other people with sub-themes and participant quotes. People were motivated by 1) specific, achievable, and challenging goals, 2) people who enjoyed their path to the goal, did not give up, achieved a goal step by step, and stayed consistent, 3) examples from people they could relate to, and 4) goal achievement itself. There are no clear differences between the conditions for the take-away responses (Fig. 6).

## Discussion

The presented study examined the use of personalized vicarious experiences in a goal-setting dialog for physical activity with a virtual coach. Our results lead to a very strong bet against people’s self-efficacy being higher after than before the dialog. This is contrary to our expectations that the dialog and the vicarious experiences therein would increase self-efficacy [2, 15]. One possible explanation for these unexpected results is the Dunning-Kruger effect [49], according to which people with little experience or knowledge regarding a task tend to overestimate their competence. Thus, after the pre-measurement, thinking about their physical activity goal and ability to achieve it may have given people more knowledge about the task. This may have led people to judge their competence more realistically, in this case lower. Similar effects have been observed by Kang et al [50] and Rowland et al [51]. More precisely, participants had lower confidence to give lectures on elementary arithmetic and lower physical activity self-efficacy, respectively, after an intervention with vicarious experiences than before.

Moreover, mirroring the findings by Kang et al [50] that the drop in self-efficacy was stronger for a condition with more personalized vicarious experience, we also saw that it is not worth betting against personalized examples leading to a higher increase in self-efficacy after the dialog than generic examples. This suggests that personalized experiences allowed people to get a more realistic picture of the task and their competence than generic ones. Interestingly, Kang et al [50] also found that the lower post-measurement of self-efficacy for the more personalized condition was more predictive of self-efficacy and perceived performance after a subsequent lecture than the post-measurement for the less personalized condition. As a more realistic assessment of one’s abilities may help to set goals that are attainable, which is important for goals to be effective [11], these findings suggest that one should not only consider how high self-efficacy is, but also how realistic the assessment of abilities is that self-efficacy is based on. Whether personalized examples indeed lead to the setting of more attainable goals is an interesting question for future work.

Comparing personalized to generic vicarious experiences with regard to their perceived motivational impact, our data lead to a promising bet that personalized experiences are more motivating than generic ones. In light of our self-efficacy findings, the motivating effect was possibly not strong enough to also influence self-efficacy positively. To increase the effectiveness of the personalization, the prediction model with a multiple $R^2$ of only 0.23 might also need improvement. This might be done by incorporating other individual characteristics such as culture [52]. Notably, however, we have already tested 22 characteristics that are in general associated with the predictors of behavior capability, opportunity, and motivation. Hence, it may be worthwhile to focus on the content of the experiences rather than individual characteristics, which can be compared to taking a content-based rather than a collaborative filtering approach to recommender systems [53]. The results of our thematic analysis of what people found motivating about the experiences they saw can serve as a basis for this.

For our last hypothesis, we saw that people’s attitude toward the virtual coach Jody is virtually certainly positive. This shows the potential of Jody for supporting people in setting effective goals, as the aspects covered in the attitude assessment such as a good relationship [54] and ease of use [55] are beneficial. However, given that people only had a single interaction with Jody, a novelty effect could have contributed to this positive attitude [56, 57].

Besides the low multiple $R^2$ of our prediction model used for the personalization of vicarious experiences, several further aspects warrant more research. First, some participants were in the precontemplation and maintenance stages of behavior change and thus either not yet interested in becoming or already physically active (Table 2). We could hence speculate that the effect of the goal-setting dialog and the personalized vicarious experiences therein is stronger for a population who are either contemplating or preparing to change. The small positive correlation between the stage of change and the acceptance of Jody provides some support for this. However, we did not find much evidence concerning the change in self-efficacy and the motivational impact ratings.

Second, personalization effects might be stronger for people who are intrinsically motivated than for people who have a monetary incentive to participate. Specifically, since social judgment theory posits that a higher degree of involvement is associated with a larger latitude of rejection in which ideas are seen as objectionable [58], intrinsically motivated people may be more likely to “object” to generic examples. Third, we only assessed the effect of the dialog and the experiences on predictors of behavior, namely, self-efficacy and reflective motivation [29]. Yet, the dialog and experiences could also affect other predictors of behavior such as automatic motivation (e.g., impulses, reflex responses) [29]. It would, therefore, be interesting to assess how behavior itself is affected. Thereby, one needs to keep in mind that it may take some time before the setting of goals affects behavior as people think about how to reach their goal [59]. Adding support for such planning (e.g., [, 60, 61]) would also be valuable, as especially people with low self-efficacy may find it difficult to come up with an effective plan [2].

## Conclusion

This study examined the effect of a goal-setting dialog and specifically personalized vicarious experiences in the context of a virtual coach for becoming more physically active. Vicarious experiences were provided by showing examples of how other people reached physical activity goals. The findings suggest that people see personalized examples as more motivating than generic ones and that people had a positive attitude toward the virtual coach. Moreover, contrary to what we hypothesized, the dialog negatively affected people’s self-efficacy. Our data also provide some support that this negative effect was stronger for the personalized examples. These findings warrant further research on how self-efficacy can be improved in combination with goal-setting dialogs. It should thereby be taken into consideration whether these lower post-measurements of self-efficacy are associated with more accurate self-assessments of abilities.

### Supplementary Information

Below is the link to the electronic supplementary material.

Supplementary file1 (PDF 557 KB)

## Appendix

### Characteristics of the people providing the examples

**Table 4:** Characteristics of the 72 people providing the examples

Characteristics	Value
Age	
- mean (SD)	42.83 (15.23)
- range	20–74
Sex	
- Female, n (%)	36 (50.00%)
- Male, n (%)	36 (50.00%)
Godin leisure time activity	
- mean (SD)	47.32 (32.14)
Household income	
- Less than £10,000, n (%)	12 (16.67%)
- £10,000–£15,999, n (%)	8 (11.11%)
- £16,000–£19,999, n (%)	6 (8.33%)
- £20,000–£29,999, n (%)	19 (26.39%)
- £30,000–£39,999, n (%)	8 (11.11%)
- £40,000–£49,999, n (%)	4 (5.56%)
- £50,000–£59,999, n (%)	5 (6.94%)
- £60,000–£69,999, n (%)	2 (2.78%)
- £70,000–£79,999, n (%)	4 (5.56%)
- £80,000–£89,999, n (%)	1 (1.39%)
- £90,000–£99,999, n (%)	1 (1.39%)
- £100,000–£149,999, n (%)	1 (1.39%)
- More than £150,000, n (%)	1 (1.39%)
Household size	
- 1, n (%)	10 (13.89%)
- 2, n (%)	11 (15.28%)
- 3, n (%)	20 (27.78%)
- 4, n (%)	21 (29.17%)
- 5, n (%)	5 (6.94%)
- 6, n (%)	4 (5.56%)
- 7, n (%)	1 (1.39%)
Personality	
- Extraversion, mean (SD)	4.17 (1.43)
- Openness to experience, mean (SD)	5.13 (1.13)
Running/walking self-efficacy$^*$	
- mean (SD)	72.98 (23.72)
Sitting hours weekend day	
- mean (SD)	6.06 (3.43)
Smoking frequency	
- Less than once per day, n (%)	5 ( 6.94%)
- At least once per day, n (%)	67 (93.06%)
TTM-stage for becoming physically active	
- Precontemplation, n (%)	2 ( 2.78%)
- Contemplation, n (%)	16 (22.22%)
- Preparation, n (%)	11 (15.28%)
- Action, n (%)	18 (25.00%)
- Maintenance, n (%)	25 (34.72%)
Weekly exercise amount	
- Less than 60 minutes per week, n (%)	24 (33.33%)
- 60–150 minutes per week, n (%)	24 (33.33%)
- More than 150 minutes per week, n (%)	24 (33.33%)

*SD* Standard Deviation, *TTM* Transtheoretical Model

*Running and walking self-efficacy were measured on scales from 0 to 100

## Individual characteristics from prediction model

**Table 5:** Individual characteristics from the linear regression model used to predict motivation ratings and how they were measured

Characteristic	Measurement
Age	Computed by Prolific based on the date of birth
Extraversion	Big-Five personality dimension [63] measured with 7-point Likert scales
Openness to experience	Big-Five personality dimension [63] measured with 7-point Likert scales
Household income	Based on the question “What is your total household in-come per year, including all earners in your household (after tax) in GBP?” on Prolific
Household size	Based on the question “Including you, how many people live in your household?” on Prolific
Physical activity identity	Based on adapting the Exercise Identity Scale by [64] to physical activity (e.g., by replacing “exercise” with “physical activity”)
Sitting hours weekend day	Based on the International Physical Activity Questionnaire (IPAQ) [65]
TTM-stage	TTM-stage for becoming physically active based on adapting the question by Norman et al [35] to physical activity using the World Health Organization’s definition of physical activity [33] and guidelines on physical activity and sedentary behavior [34]
Godin leisure time activity level	Based on Godin [66]
Running/walking self-efficacy	Based on an adaptation of the Exercise Self-Efficacy Scale by citetmcauley1993 self (see Supplementary Information 1)

*TTM* Transtheoretical Model

## Generic examples

**Table 6:** The generic examples: People in the “generic” condition received two of the three overall most motivating examples. Each example consists of an introduction of the example person as well as a description of the goal and how the example person achieved the goal

Introduction	Goal and How They Achieved It
I enjoy the outdoors. My hobbies are building puzzles and riding horses. I’m easy to talk to and I listen well.	I have walked more than 10.000 steps in half a day. I achieved this by walking everywhere I needed to be.
Hello, I like to meet new people. One of my hobbies is watching tv. I am trying to stay in shape by walking.	I set a goal to walk every day regardless of weather factors for 1 straight month, and I achieved the goal. I achieved this by setting all my feelings aside and forced myself to ensure I met this goal.
I started having walks around country lanes which is nicer than walking alone on a road. I prefer to look at sheep and cows rather than cars and buses.	I increased the time that I am walking by walking further. I achieved this by building up my walks by doing a little bit more each month.

## Correlations between outcome measures and stage of change

**Table 7:** Results of Bayesian analyses of Pearson correlations between the TTM-stage for becoming physically active on the one hand and the outcome measures of change in self-efficacy between the pre- and post-measurement, motivational impact ratings for the two types of examples, and the acceptance on the other hand

Outcome Measure	Mean (SD)	95% CI	Post > 0	Evaluation
Change in self-efficacy	0.00 (0.16)	[-0.31, 0.32]	0.49	Not worth betting against
Motivational impact generic examples	0.04 (0.16)	[-0.28, 0.36]	0.60	Not worth betting on
Motivational impact personalized examples	0.04 (0.17)	[-0.28, 0.37]	0.59	Not worth betting on
Acceptance	0.22 (0.16)	[-0.08, 0.53]	0.92	A promising but risky bet

*TTM* Transtheoretical Model, *SD* Standard deviation, *CI* Credible interval

Post > 0, Posterior probability that the correlation is greater than 0

## Footnotes

1 We randomly chose one example for each of the 18 combinations of values for sex (2 values), age range (3 values), and weekly exercise amount (3 values) from the people providing the examples.

2 See Table 5 for how these individual characteristics were measured. Details on the individual items can be found in the provided dataset.

3 We used the smoking status as one balancing factor since Jody was developed as part of the Perfect Fit project [38], which aims to develop an eHealth application for quitting smoking and becoming more physically active.
