# Supplementary material for: Setting Physical Activity Goals with a Virtual Coach: Vicarious Experiences, Personalization and Acceptance
Source: J Med Syst. 2023 Jan 30;47(1):15. doi: 10.1007/s10916-022-01899-9 (PMC9884656; doi:10.1007/s10916-022-01899-9)
Supplement: Supplementary file 1 — Supplementary file1 (PDF 557 KB) [file 10916_2022_1899_MOESM1_ESM.pdf]

Albers, N.*, Hizli, B., Scheltinga, B.L., Meijer, E., and Brinkman, W.P. (2023). Setting physical activity goals with a virtual coach: Vicarious experiences, personalization and acceptance. *Journal of Medical Systems*.

*Intelligent Systems, Delft University of Technology, Delft, The Netherlands
E-mail: n.albers@tudelft.nl

Running Self-Efficacy Questionnaire

Using the scales listed below please indicate how **confident** you are that you will be able to **run at a moderate intensity** for the given amount of minutes **per week**. When you're running at a moderate intensity, you breathe heavily but you can still hold a short conversation.

	Not at all confident — Moderately confident — Highly confident<br>0 10 20 30 40 50 60 70 80 90 100
How confident are you to run for **15** minutes per week? ()	
How confident are you to run for **30** minutes per week? ()	
How confident are you to run for **45** minutes per week? ()	
How confident are you to run for **60** minutes per week? ()	
How confident are you to run for **75** minutes per week? ()	
How confident are you to run for **90** minutes per week? ()	
How confident are you to run for **105** minutes per week? ()	
How confident are you to run for **120** minutes per week? ()	
How confident are you to run for **135** minutes per week? ()	
How confident are you to run for **150** minutes or more per week? ()	

The mean of all confidence ratings is used as an index measure.
